# Evidence summary of meaning in life intervention for cancer patients

**DOI:** 10.3389/fonc.2025.1603349

**Published:** 2025-07-30

**Authors:** Ping Yuan, Weinan Lu, Xia Yong, Wenli Yang, Xiaoying Zhong, Meng Wang, Yiying Zhang, Ting Fang, Yan Xie, Xuemei Li, Limei Zhang

**Affiliations:** ^1^ School of Nursing, Chengdu Medical College, Chengdu, China; ^2^ School of Clinical Medicine, Chengdu Medical College, Chengdu, China; ^3^ Department of Gastroenterology, The First Affiliated Hospital of Chengdu Medical College, Chengdu, China; ^4^ People’s Hospital of NANBU County, Nanchong, China; ^5^ The General Hospital of Western Theater Command, Chengdu, China; ^6^ Department of Infection Division, The First Affiliated Hospital of Chengdu Medical College, Chengdu, China; ^7^ Sichuan Provincial Key Laboratory of Philosophy and Social Sciences for Intelligent Medical Care and Elderly Health Management, Chengdu, China

**Keywords:** cancer patients, meaning of life, evidence summary, evidence-based nursing, cancer

## Abstract

**Objective:**

To evaluate and summarize evidence on intervention of the meaning of life of cancer patients, and provide evidence-based basis for clinical practice.

**Methods:**

According to the “6S” evidence model, The literature related to the life meaning intervention in cancer patients were systematically searched in domestic and foreign evidence-based resource databases,comprehensive databases and professional society websites from the inception of database to Oct 2023. Two researchers evaluated the quality of the literature, extracted and integrated evidence.

**Results:**

A total of 28 articles were included, including 2 the computer decisions, 4 guidelines, 7 systematic evaluations, 6 quasi-experimental studies, and 9 randomized controlled trials. A final total of 48 pieces of evidence were summarized, including 5 areas of organizational management, assessment, evaluation indicators, intervention programs for meaning of life, and intervention techniques.

**Conclusion:**

This study forms the evidence of intervention of the meaning of life of cancer patients, which can provide reference for clinical practice, Individualized treatment and nursing care should be provided according to the symptoms and actual needs of the patients, and relevant evidence should be updated in time.

## 1 Introduction

Cancer is now the second leading cause of death worldwide, with the number of deaths and cases increasing every year (1). The number of new cancer cases worldwide is expected to reach 28.4 million by 2040, an increase of 911 million (47%) from 2020, according to the related study (2). In recent years, various modalities such as radiotherapy, chemotherapy, surgery, targeted therapy, and Immune therapy have been employed in the comprehensive management of tumors, contributing to an extension of patient survival. Nevertheless, the pain and suffering associated with the disease may still leave patients feeling physically and mentally fatigued while exacerbating negative emotions like anxiety and depression. This can instigate feelings of fear and insecurity, ultimately diminishing or eroding one’s sense of life’s meaning and potentially leading to suicidal thoughts (3). The meaning of life was first proposed by FANK, which refers to the individual’s perception and feeling of the meaning and purpose of life. Its connotation is the need to explore and understand the purpose and meaning of life when an individual’s life is faced with danger or even defeat (4). In positive psychology, meaning in life is closely related to mental health, which can promote physical and mental health, give individuals a stronger ability to resist stress, improve happiness, and promote a positive attitude to life (5). Simultaneously, the significance of meaning in life serves as a crucial determinant for fostering spiritual and psychological well-being, as well as enhancing overall quality of life, Zhang and Jin showed that there was a significant negative correlation between meaning in life and negative mental health indicators through meta-analysis (6). Studies have shown that meaning in life can reduce suffering, and even reduce or prevent the emergence of beliefs that “desire to accelerate death” in people (7). some scholars pointed out that helping patients find the meaning of life is the responsibility of nurses (8–12). At present, a number of meaning-centered interventions have been carried out abroad, It is confirmed that in the process of pursuing the meaning of life, patients’ sense of hope and desire for survival are significantly increased, and their emotional anxiety, depression and fear are significantly reduced, so that patients with advanced cancer can be more calm in the face of death. It is helpful to help them find spiritual peace, happiness and meaning, so as to significantly improve their quality of life (13–15). The existing clinical evidence for the intervention of meaning in life of cancer patients in China is relatively scattered, and it lacks fine design and does not fully reflect the personalized element. Therefore, this study systematically retrieved the research on the life meaning intervention of cancer patients at home and abroad, The method of evidence-based nursing was used to evaluate and integrate the evidence, so as to provide a reference for medical staff to implement the personalized nursing plan for the meaning of life.

## 2 Materials and methods

### 2.1 Establish evidence-based issues

The PIPOST model developed by the Evidence-based Nursing Center of FUDAN University was used to propose structured evidence-based questions (16).The target Population of evidence application is cancer patients; Intervention is a series of measures to improve the meaning of life of cancer patients; Evidence implementation professionals are clinical caregivers; The outcome indicator is the improvement of the meaning of life; The Setting for evidence application is the clinical department with tumor patients; The types of evidence included clinical decisions, practice guidelines, systematic reviews, various original studies. This study has been registered on the Evidence Summary registration platform of the Evidence-based Nursing Center of FUDAN University (Registration No: ES20220915).

### 2.2 Retrieval strategy

Firstly, based on the “6S” pyramid model, through the combination of subject words and free words, top-down searches were conducted on system, summaries, synopses of syntheses, synthesis, synopses of studies, and studies (17). Literature and data extraction were screened based on the inclusion and exclusion criteria of evidence-based questions constructed by PIPOST. Then evaluate the quality of the literature, extract the evidence and classify the evidence, and finally summarize to form the evidence summary.

English search terms “ tumor* OR cancer* OR malignancy* OR neoplasm* ” AND “ ‘meaning in life’ OR ‘meaning of life’ OR ‘life meaning’ OR ‘the meaning of life’ OR ‘Sense of meaning in life’ ” AND “guideline* OR consensus OR review* OR meta OR analysis OR study OR practice OR research OR trial”. Computer searches Up To Date, Joanna Briggs Institute (JBI), National Comprehensive Cancer Network (NCCN), Guidelines International Network (GIN), MEDLIVE, BMJ Best Practice, National Guideline Clearinghouse (NGC), New Zealand Guidelines Group (NZGG), Scottish Intercollegiate Guidelines Network (SIGN), Australian Clinical Practice Guidelines, CMA INFOBASE, WHO, Registered Nurses’ Association of Ontario (RNAO), Web of Science, COCHRANE Library, EMBASE, PUBMED, Ovid, EBSCO, WANFANG Database, China National Knowledge Infrastructure (CNKI), China Biology Medicine disc (CBM), Institute for Clinical Systems Improvement (ICSI), European Society for Medical Oncology (ESMO), World Hospice Palliative Care Alliance (WHPCA), International Society for Hospice and Palliative Care (IAHPC), Canadian Association for Psycho-social Oncology (CAPO), National Council Palliative Care (NCPC), Irish Hospice Foundation, Japanese Society for Palliative Medicine (JSPM), European Association for Palliative Care (EAPC), National Hospice and Palliative Care Organization etc. comprehensive database. The literature was searched for publications from the establishment of the database to Oct 2023.

### 2.3 Literature inclusion and exclusion criteria

Inclusion Criteria: (i) The types of literature were best practices, guidelines, expert consensus, systematic reviews, and the latest randomized controlled trials that were not included in the above evidence-based resources, and evidence-based resources such as systematic reviews and guidelines were the latest versions; (ii) The content of the literature included the meaning of life intervention for cancer patients; (iii) Published Chinese and English literature. Exclusion Criteria: (i) Translation the Chinese version of foreign literature; (ii) Literature with incomplete data or data that could not be extracted; (iii) Conference Papers; (iv) Duplicate published literature; (v) Scientific instruction manual; (vi) Relevant discussion drafts, conference abstracts, excerpts, interpretations, and draft guidelines specified in the guidelines.

### 2.4 Literature screening and data extraction

Two researchers with systematic evidence-based training independently screened the literature for inclusion and exclusion criteria by reviewing the titles, abstracts, and full texts. They extracted basic information and data from the selected studies and cross-checked the results. Any discrepancies were resolved through discussion with a third researcher to reach a consensus.

### 2.5 Literature quality evaluation

Two researchers, who are Master’s students trained in a systematic evidence-based program, independently evaluated and graded the quality of evidence for inclusion. If any disagreements arise, a third investigator was sought to participate in decision-making and proofread the translation of the English evidence. 1) Guidelines: Guidelines were evaluated using the Appraisal of Guidelines for Research and Evaluation II (AGREE II) (18), which includes six domains: scope objectives, personnel involved, and development rigor, with 23 entries each representing a score of 1–7 from “strongly disagree” to “strongly agree”. Scores were standardized to the highest possible percentage of scores in the domain. 2) Systematic reviews or Meta-analysis: the quality of the evaluation was assessed using the Assessment of Multiple Systematic Reviews II (AMASTAR-2) tool (19), 3) Expert consensus and experimental research: the evaluation criteria developed by the JBI Center for Evidence-Based Health Care in its 2016 edition was used (20), which included six entries to label sources of opinion, reference to other literature, and state conclusions.

### 2.6 Evidence extraction, integration and evaluation

The content analysis method was used to extract evidence from the literature, which included general characteristics, research themes, and main contents of the literature. When evidence from different sources had complementary or consistent conclusions, a combined or general expression was used. However, if there were conflicting evidence from different sources, the principles of evidence-based priority, high-quality evidence priority, and latest published authoritative literature priority were followed. We graded the aggregated evidence using the Joanna Briggs Institute (JBI) Levels of Evidence and Grades of Recommendation system (2014 version) (21) from the Australian JBI Centre for Evidence-Based Healthcare. This system categorizes the evidence into five levels, from high to low, based on the study design of the included literature.

## 3 Results

### 3.1 Selection process and general characteristics of included literature

**Figure 1:**
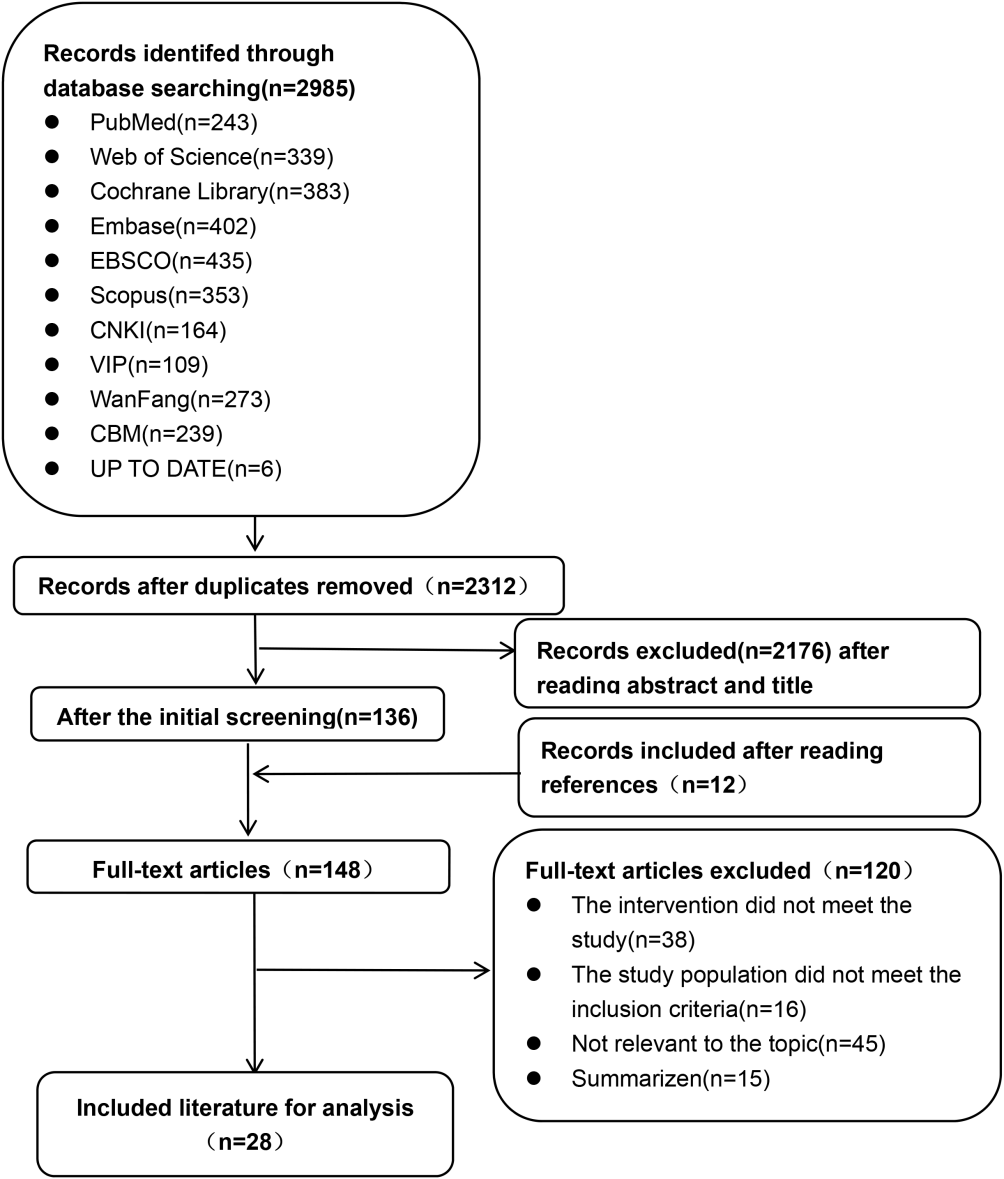
A flow chart of the literature screening process.

An initial search yielded a total of 2,985 articles, which were reduced to 673 after removing duplicates. Following a review of titles, abstracts, and full texts to eliminate non-compliant literature, In total, 28 articles were ultimately included in the study, consisting of 4 guidelines, 2 Computerized decision support systems, 6 quasi-experimental studies, 9 randomized controlled trials, and 7 systematic reviews. **Table 1** provides an overview of the basic characteristics of the literature included, while **Figure 1** shows a flowchart detailing the literature screening process.

**Table 1:** Characteristic of included literatures (n=28).

Included literatures	Year	Source	Type of evidence	Topic
Meyer (44)	2021	UP TO DATE	Computerized decision support systems	Psychosocial aspects of advanced illness
Breitbart (46)	2022	UP TO DATE	Computerized decision support systems	Assessment and management of depression in palliative care
Riba (42)	2023	NCCN	Guideline	Management of psychological distress
Howell (47)	2012	CAPO	Guideline	Mental Health Nursing Needs Assessment for Adult Cancer Patients
Brundage (43)	2020	ICSI	Guideline	Palliative care for adults
Crawford (45)	2021	ESMO	Guideline	End-of-life care for adults with cancer
Vos (24)	2015	Web of Science	Systematic review	Meta-analysis of the effects of existential therapy on psychological outcomes
Wang (22)	2017	PubMed	Systematic review	Effects of life review interventions on mental health, psychological distress, and quality of life in patients with advanced or terminal cancer: a systematic review and Meta-analysis of randomized controlled trials
Kang (15)	2019	PubMed	Systematic review	Meta-analysis of Meaningful Center Interventions for Patients with Advanced or Late-Stage Cancer
Tuominen (23)	2019	PubMed	Systematic review	Systematic evaluation of the effectiveness of nursing interventions for cancer patients
Chu (25)	2020	Web of Science	Systematic review	How Positive Thinking Enhances the Meaning of Life:A Meta-Analysis of Related Studies and Randomized Controlled Trials
Manco (13)	2021	PubMed	Systematic review	Meta-analysis of interventions to promote meaning in life
Wang (26)	2023	PubMed	Systematic review	A systematic review of interventions for demoralization in cancer patients
CHEN Mingjin (36)	2013	CNKI	Quasi-experimental study	Establishment of localized meaning therapy and preliminary evaluation of its therapeutic effect on advanced cancer patients
LI Jianghua (37)	2018	CNKI	Quasi-experimental study	The effect of meaning therapy on life attitudes and quality of life in patients with advanced breast cancer
Chochinov (38)	2005	PubMed	Quasi-experimental study	Dignity therapy: a novel psychotherapeutic intervention for the terminally ill
Kang (39)	2015	Scopus	Quasi-experimental study	Development and Initial Testing of a Meaning-Centered Youth Program for Advanced Cancer
Datta (40)	2016	PubMed	Quasi-experimental study	Potential utility of Acceptance and Commitment Therapy (ACT) to reduce stress and improve well-being in cancer patients in Kolkata
Gomez-Batiste (41)	2017	PubMed	Quasi-experimental study	Strengthening psychosocial and spiritual palliative care: four-year results of a comprehensive care program for patients with advanced disease and their families in Spain
Ming (27)	2017	CNKI	Randomized controlled trial	A study of the construction and application effects of a meaning-of-life intervention program for advanced cancer patients
Zhu (28)	2020	WanFang	Randomized controlled trial	Effectiveness of Meaningful Treatment in Patients with Advanced Lung Cancer
Zhang (29)	2022	WanFang	Randomized controlled trial	The effect of narrative care on meaning and quality of life in patients undergoing chemotherapy for advanced lung cancer
Hsiao (34)	2012	PubMed	Randomized controlled trial	Effect of psychotherapy on mental health and circadian cortisol levels in breast cancer patients
Piderman (35)	2014	PubMed	Randomized controlled trial	Spiritual quality of life in patients treated with radiation for advanced cancer
Chen (30)	2020	PubMed	Randomized controlled trial	The effect of a mind-mapping-based life review program on the psychospiritual well-being of cancer patients receiving chemotherapy: a randomized controlled trial
Holtmaat (33)	2020	Cochrane Library	Randomized controlled trial	Long-term efficacy of meaning-centered group psychotherapy for cancer survivors: 2-year follow-up results of a randomized controlled trial
Teskereci (31)	2022	Embase	Randomized controlled trial	Impact of a nursing program based on human caring theory on gynecologic cancer patients: a pilot study from Turkey
Bower (32)	2022	PubMed	Randomized controlled trial	Improving biobehavioral health in young breast cancer survivors: pathways to secondary outcomes in health trials

### 3.2 Quality evaluation results of the included literature

#### 3.2.1 Quality evaluation results of the computerized decision support systems

Two Computerized decision support systems were included in this study. Clinical decision making is the highest quality evidence for direct inclusion in this study.

#### 3.2.2 Quality evaluation results of the guidelines

Four guidelines were included in this study. The guidelines were evaluated using AGREE II and the results are shown in **Table 2**, of which three were A-recommendations and one was a B-recommendation. The overall quality was high and inclusion was granted.

**Table 2:** Results of methodological quality evaluation included in the guidelines.

Guidelines	Percentage of field standardization %						≥60% field number (n)	≥30% field number (n)	Recommendation level
	Scope and purpose	Participant	Rigor	Clarity	Application	Independence			
Howell (47)	100.00	81.94	79.16	95.83	67.71	95.83	6	6	A
Brundage (43)	100.00	95.83	98.44	98.61	82.29	100.00	6	6	A
Crawford (45)	91.84	87.50	67.71	98.61	67.74	95.83	6	6	A
Riba (42)	100	88.89	77.08	95.83	65.63	29.2	5	6	B

#### 3.2.3 Quality evaluation results of systematic reviews

Seven systematic reviews were included in this study. In the study by Kang et al (15) and Wang et al (22). “Did the report of the review contain an explicit statement that the review methods were established prior to the conduct of the review and did the report justify any significant deviations from the protocol?”, “Did the review authors use a comprehensive literature search strategy?” were evaluated as “partially yes” and “Did the review authors report on the sources of funding for the studies included in the review?” was evaluated as “no”, the rest of the evaluation results were “yes”. In the study by Tuominen et al (23). “Did the report of the review contain an explicit statement that the review methods were established prior to the conduct of the review and did the report justify any significant deviations from the protocol?” was evaluated as “partially yes”, “Did the review authors report on the sources of funding for the studies included in the review?”, “If meta-analysis was performed, did the review authors use appropriate methods for statistical combination of results?” were evaluated as “no” and “if meta-analysis was performed, did the review authors assess the potential impact of Rob in individual studies on the results of the meta-analysis or other evidence synthesis?” was evaluated as “Meta-analysis was not performed”, while all other entries were evaluated as “yes”. In the study by Vos et al (24). “Did the report of the review contain an explicit statement that the review methods were established prior to the conduct of the review and did the report justify any significant deviations from the protocol?” was evaluated as “partially yes” and “Did the review authors report on the sources of funding for the studies included in the review?” was evaluated as “no*”*, the rest of the entries were evaluated as “yes”. In the study by Chu et al (25). “Did the report of the review contain an explicit statement that the review methods were established prior to the conduct of the review and did the report justify any significant deviations from the protocol?”, “Did the review authors use a comprehensive literature search strategy?” were evaluated as “partially yes”, the rest of the evaluation results were “yes”. In the study by Manco et al (13). “Did the report of the review contain an explicit statement that the review methods were established prior to the conduct of the review and did the report justify any significant deviations from the protocol?”, “Did the review authors provide a list of excluded studies and justify the exclusions?”, “Did the review authors describe the included studies in adequate detail?” were evaluated as “partially yes” and “Did the review authors report on the sources of funding for the studies included in the review?” was evaluated as “no”, the rest of the entries were evaluated as “yes”. In the study by Wang et al (26). “Did the review authors perform data extraction in duplicate?”, “Did the review authors report on the sources of funding for the studies included in the review?”, “If meta-analysis was performed, did the review authors use appropriate methods for statistical combination of results?” were evaluated as “no”, “Did the review authors use a comprehensive literature search strategy?” was evaluated as “partially yes” and “If meta-analysis was performed, did the review authors assess the potential impact of Rob in individual studies on the results of the meta-analysis or other evidence synthesis?” was evaluated as “Meta-analysis was not performed”, the rest of the evaluation results were “yes”. The articles were all included.

#### 3.2.4 Quality evaluation results of randomized controlled trials

A total of 9 randomized controlled trials were included in this study. the study by Ming et al (27). entry 2“Was allocation hidden?”, entry 4*“*Were the subjects blinded?”, entry 5“Was the intervention blinded?”, entry 6“Were outcome evaluators blinded?” were evaluated as“unclear”, entry 8“Was follow-up complete, and were measures taken to manage loss to follow-up?”, entry 9“Were all randomly assigned subjects included in the outcome analysis?” were evaluated as “no”, the rest of the evaluation results were “yes”. In the study by Zhu et al (28). entry 2“Was allocation hidden?”, entry 4“Were the subjects blinded?”, entry 6*“*Were outcome evaluators blinded?” were evaluated as “unclear”, entry 5 “Was the intervention blinded?” was evaluated as “no”, the rest of the entries were evaluated as “yes”. In the study by Zhang et al (29). entry 2“Was allocation hidden?”, entry 4“Were the subjects blinded?”, entry 6“Were outcome evaluators blinded?” were evaluated as “unclear”, entry 5“Was the intervention blinded?”, entry 8“Was follow-up complete, and were measures taken to manage loss to follow-up?”, entry 9“Were all randomly assigned subjects included in the outcome analysis?” were evaluated as “no”, the rest of the entries were evaluated as “yes”. In the study by Chen et al (30). All entries were evaluated as “yes”, except for entry 4“Were the subjects blinded?” and entry 5“Was the intervention blinded?”, which were evaluated as “no”. In the study by Teskereci et al (31). entry 5“Was the intervention blinded?” and entry 6“Were outcome evaluators blinded?” were evaluated as “no”, while all other entries were “yes”. For the study by Bower et al (32). entry 1”Were the subjects really randomized?”, entry 2“Was allocation hidden?”, entry 4“Were the subjects blinded?”, entry 5“Was the intervention blinded?”, entry 6“Were outcome evaluators blinded?” were evaluated as “unclear”, entry 3“Were the groups comparable at baseline?” was evaluated as “no”, the rest of the entries were evaluated as “yes”. For the study by Holtmaat et al (33) and Hsiao et al (34). entry 2“Was allocation hidden?”, entry 4”Were the subjects blinded?”, entry 5“Was the intervention blinded?”, entry 6“Were outcome evaluators blinded?” were evaluated as “unclear”, entry 9“Were all randomly assigned subjects included in the outcome analysis?” was evaluated as “no”, the rest of the evaluation results were “yes”. For the study by Piderman et al (35). entry 1*“*Were the subjects really randomized?”, entry 2 “Was allocation hidden?”, entry 4“Were the subjects blinded?”, entry 5“Was the intervention blinded?”, entry 6“Were outcome evaluators blinded?” were evaluated as “unclear”, entry 9“Were all randomly assigned subjects included in the outcome analysis?” was evaluated as “no”, the rest of the evaluation results were “yes”. The study design was relatively complete, and the overall quality was moderate, and was included after discussion by the study team.

#### 3.2.5 Quality evaluation results of quasi-experimental studies

6 quasi-experimental studies were included in this study. For the studies by Chen et al (36), LI et al (37) and Chochinov et al (38). all the entries were “yes”. For the studies by Kang et al (39) and Datta et al (40). all the entries were “yes” except 6“Was follow-up complete, and was loss to follow-up reported and managed?” was evaluated as “no”. In the study by Gomez-Batiste et al (41). “Was follow-up complete, and was loss to follow-up reported and managed?” was evaluated as “unclear”, while all other entries were evaluated as “yes”. The articles were all included.

### 3.3 Summary of the evidence

The evidence related to the 28 included literature will be extracted and summarized by this research team, resulting in a synthesis of evidence from five areas of Organization and management, evaluation, evaluation indicators, programs to improve the meaning of life, intervention skills and precautions, resulting in 48 pieces of evidence, with the aim of providing healthcare professionals with better guidance for improving meaning in the life of cancer patients provide an evidence-based basis in **Table 3**.

**Table 3:** Summary of the evidence.

Category	Evidence content	Evidence level	Recommen dation level
Organization and management	1. Interdisciplinary collaboration is recommended (42, 43).	1c	A
	2. Ideally, membership should include executive leadership, physicians, advanced practice providers,nurses,social workers,information technology specialists,spiritual counselors,psychologists,pastors, bereaved persons (35, 41–44).	1c	A
	3. Nurses are the most common intervention providers (23, 41).	1b	A
	4. Members involved in the intervention need ongoing training in relevant professional theory and knowledge (26, 43).	1b	A
	5. It is recommended that the medical staff hold ongoing care conferences with patients,families, and members of the interdisciplinary team (43).	1c	A
evaluations	6. Meaning of life assessment and screening is recommended for cancer patients (42–44).	1c	A
	7. Psychiatrists, psychologists, nurses, advanced practice clinicians, or social workers can perform mental health assessments and treatments (42).	2a	A
	8. At key points in the cancer continuum assessment: initial diagnosis,initiation of treatment,periodic intervals during treatment,end of treatment, post-treatment or transition to survival, recurrence or progression, advanced disease, at death, and during an individual's transition or reassessment in the midst of a family crisis, during survivorship, near death (43).	5b	B
	9. Use a validated assessment tool to assess (43).	1c	A
Evaluation indicators	10. Meaning of life scales used abroad (23): the FACIT-Sp meaning subscale, the Quality of Life Issues at the End of Life scale, the Purpose in Life scale, the Life Orientation Test using the LOT scale, the Crumbaugh scale, and the Existence subscale from the McGill Quality of Life questionnaire, and the Life Attitude Scale (31).	1b	B
	11. Life Attitude Profile Scale adapted by Yingqi He et al (37).	2d	A
	12. The Meaning of Life Scale for Advanced Cancer Patients developed by Yongsheng Wu is the current domestic scale for measuring the meaning of life for advanced cancer patients (27, 29).	1c	A
	13. Other: mental health, quality of life, anxiety, depression and physical symptoms etc (15).	1b	B
Intervention programs that enhance the meaning of life	14. Dignity therapy (14, 26, 38, 43, 45): a psychosocial intervention that helps patients maintain and improve their sense of dignity and enhance their sense of meaning and value in life by reviewing important events, achievements, etc. in their lives.	1b	A
	15. Meaning therapy (13, 15, 24, 26–28, 33, 36, 39, 42, 45, 46): focuses on guiding patients to search for and discover the meaning of life, so that they can change their attitudes and ways of life, especially suitable for those who are facing disasters or in crisis.	1a	A
	16. Life Review Therapy (22, 43, 46): Helps patients discover new meaning and improve the quality of life by reviewing and reorganizing life experiences.	1a	A
	17. Advance Care Planning (43): The process by which an individual, while conscious, clearly expresses in advance the type of medical treatment and care he or she would like to receive in the event of a	4d	B
	serious health or life-threatening condition.		
	18. Positive Thinking Therapy (13, 25, 32, 40, 42, 45): centers on positive thinking by consciously being aware of the present moment and confronting current feelings, emotions, and thoughts without any judgment.	1a	A
	19. Narrative therapy (13, 29, 45): Through the methods of “storytelling” and “externalization of problems”, helping individuals to reconstruct their own stories, encouraging them to look at problems from a new perspective, and getting rid of negative emotions and difficulties.	1a	A
	20. Music therapy (45): music influences a person's emotional, behavioral and physiological responses, allowing people to gain a deeper understanding of their inner needs and values.	2a	B
	21. Psychological care treatment (13, 23): In the nursing process, by the nursing staff through various ways and means, to improve or eliminate psychological problems and behaviors, to obtain the most appropriate physical and mental state.	1a	B
	22. Body-Mind-Spirit Group Therapy (34): blends Chinese and Western medical and meaning-of-life philosophies to enhance patients' stress coping skills and resilience by alleviating suffering and providing holistic empowerment strategies that view cancer as an opportunity for personal growth.	1c	B
	23. Structured multidisciplinary intervention (35): a structured reflection on topics related to the material, emotional, social and spiritual domains, moderated by a professional and involving multidisciplinary staff, with the aim of improving the mental quality of life of cancer patients.	1c	A
	24. Mind mapping-based life review program (30): using mind maps to guide an entire session or facilitate a review of a specific negative event, participants are guided to explore life and death.	1c	B
	25. A care plan based on the theory of human caring (31): holistic, individualized care based on the theory of human caring that supports the patient's hope, helps him or her find meaning in life, and strengthens his or her ability to cope with cancer.	1c	A
Tips and Cautions in Intervention	26. Effective management of symptoms such as pain increases the opportunity for patients to participate in meaningful activities (44).	4b	A
	27. Clarify the patient's and family's values and feelings about the disease through proper questioning and dialog, communicating the most optimistic hopes and taking into account feared outcomes, and recognizing spiritual or existential suffering (44).	5b	B
	28. Compassionate listening skills can be used to understand the patient's pain (45).	5c	A
	29. Apply family theory and clinical experience to support the value of assessing family functioning and identifying important relationships (44).	1c	A
	30. Prayer is the main help taken by many patients (42).	2a	B
	31. Career fulfillment and parenting as a way for people with advanced cancer to discover the meaning of life (30).	1c	A
	32. It is recommended that humanism and existentialism be combined in the care of oncology patients (31).	1c	A
	33. Integrating psychosocial support with existing outpatient palliative care services is a proven model (41).	2d	B
	34. Guiding cancer patients' thinking about the realm of existence, such as asking about existential concepts such as life, death, and the purpose of living can increase their discovery of the meaning of life and increase the patient's ability to find meaning in life (31).	1c	A
	35. Convey a caring attitude, focus on achievable short-term goals (44).	5b	A
	36. Informed consent should be obtained from patients with decision-making capacity or appropriate surrogate decision makers prior to intervention (43).	3e	A
	37. Evaluation and treatment should occur early in the disease (45).	1a	A
	38. For in-depth assessment and counseling, referral should be made to a trained chaplain or spiritual care professional (42, 45).	2a	A
	39. Interventions should not place an additional burden on patients (23).	1b	A
	40.The number of interventions is critical, with a minimum of three interventions required for maximum benefit (26, 41).	1b	B
	41.The long-term impact of the intervention on the patient also needs to be considered, especially for those who have survived cancer for a long time (26).	1b	B
	42.Cancer patients were more likely to participate in psychosocial interventions delivered over the phone than face-to-face (42).	2a	B
	43.Clinical interventions for cancer patients should be individualized (26).	1b	A
	44.Individual approach is the most common form of treatment, other forms include individual and group combination, etc (22).	1b	A
	45.Advocate for group interventions for cancer populations with low symptom burden (26).	1b	A
	46.Psychosocial interventions targeting patients and their families are more effective than individual interventions (42).	2a	A
	47.The impact of meaning-centered interventions on meaning in life was homogeneous, with no significant differences in individual, group intervention, or session frequency (15).	1b	A
	48.The magnitude of the effect of positive thinking on meaning is consistent across nationalities and across countries (25).	1a	B

## 4 Discussion

This study summarized the evidence related to meaning in life intervention for cancer patients in five areas, including Organization management, evaluation, evaluation indicators, intervention programs that can improve the meaning of life, intervention skills and precautions.

### 4.1 Analysis of intervention measures for the meaning of life of cancer patients

A systematic literature search revealed that, At present, the interventions carried out at home and abroad to improve the meaning of life of cancer patients include dignity therapy, logo therapy, life review therapy, advance care planning, mindfulness therapy, narrative therapy, music therapy, psychological care, body-mind-spirit group therapy, multidisciplinary structured intervention, life review plan based on mind map, and nursing based on humanistic care theory Plans, and so on. Domestic meaning in life intervention research started late, and the depth and breadth of research are still limited. In China, there are mainly dignity therapy, logo therapy, life review therapy, mindfulness therapy, and narrative therapy. There were no superior or inferior interventions, and there was concern about the accuracy of generalizations about the strengths and weaknesses of different interventions. Since most of the intervention data come from abroad, there may be some differences with the cultural background of our country. Sociocultural adaptability is a key consideration for the localized implementation of intervention measures. Therefore, future research needs to summarize the previous evidence, combine with China’s cultural background, cognitive level, disease stage and cultural values to formulate a meaning in life intervention program for cancer patients suitable for China, and study and verify the feasibility and effectiveness of the intervention measures to ensure its practical value in the local environment.

### 4.2 Effect of intervention measures on meaning of life in cancer patients

Studies have shown that interventions based on the meaning of life can increase the inner peace of cancer patients, improve negative emotions such as anxiety, depression, and fear, help patients perceive and find meaning in life, maintain and improve the meaning of life, and reduce despair at the end of life. However, there is a lack of clear rankings of post-intervention effect sizes and specific recommendations for these interventions.It is recommended to analyze the effect size of different interventions on the improvement of the meaning of life of cancer patients through network Meta-analysis, and screen out the most effective intervention. For the long-term effect after intervention, multi-center collaborative longitudinal follow-up research can be carried out in the future, and electronic health databases or mobile health websites can be used to reasonably formulate research programs and coping strategies for loss to follow-up, and quantitative and qualitative research can be combined to comprehensively understand the changes in the meaning of life of patients.

### 4.3 Multidisciplinary collaborative teams take an active role in meaningful life management for cancer patients

Research has shown that the study of life meaning interventions for cancer patients is multidimensional and involves multiple disciplines such as psychology, medicine, and sociology. Not only does it require in-depth exploration of changes in the patient’s psychological state, physiological responses, social support network, and life values, but it also requires the effective integration of these different dimensions of knowledge to develop more individualized, comprehensive, and effective interventions. However, the current interdisciplinary cooperation in China is not close enough, resulting in limited research horizons and methods. Therefore, it is recommended that in the future, all disciplines should strengthen their cooperation and work together to promote the progress and development of meaningful life interventions for cancer patients, so as to provide patients with more comprehensive, precise and humanistic services.

To promote the implementation and effectiveness of meaning in life interventions for cancer patients, we need to work together from many aspects. Therefore, Interdisciplinary collaboration has thus become a key pathway for advancing the field (42, 43). Firstly, the active participation and recognition of professionals is the key to the success of meaning in life intervention. Ideally, membership should include executive leadership, physicians, advanced practice providers, nurses, social workers, information technology specialists, spiritual counselors or psychologists or chaplains, bereaved persons (35, 41–44). Professionals should give full play to their professional advantages, establish a trust relationship with patients, provide effective psychological support and guidance, enhance patients’ psychological resilience and stress resistance, so as to enhance patients’ meaning in life. At the same time, the professional competence of medical staff directly affects the effect of meaning in life intervention. Therefore, it is necessary to strengthen the mental health knowledge training of medical staff to improve their understanding and coping ability for mental health problems. Medical staff should fully respect the wishes and choices of patients, convey positive and optimistic attitudes and emotions, give patients confidence and hope, and provide personalized support and services according to their individual needs when carrying out meaning in life intervention. Interventions can achieve better results only if they truly focus on the needs and wishes of patients.

In order to enhance the sustainability and effect of intervention, we can understand the economic situation of patients and provide corresponding financial assistance and social support according to their needs to help them overcome economic difficulties, and at the same time, let patients develop a variety of interests to improve their quality of life and meaning of life (45). Unilateral power is limited. We need to establish interdisciplinary teams composed of professionals with different professional backgrounds. Cooperation and coordination among team members can improve the quality and effect of services, so that patients can receive better support and help (46). In addition, we also need policy support and attention from the leadership of the unit. It is necessary to strengthen the formulation and implementation of relevant policies, provide necessary resources and support, and promote the in-depth development of meaning in life intervention.

### 4.4 Meaning of life assessment and care cannot be ignored

Studies have shown that early assessment and screening of cancer patients’ mental health, quality of life, anxiety, depression, and physical symptoms facilitates healthcare professionals to have a timely and comprehensive understanding of the patient’s current status in order to develop individualized interventions for the patient (42–44). The assessment should be carried out using a validated assessment tool (43). The person conducting the assessment may be a psychiatrist, psychologist, nurse, advanced practice clinician, or social worker, and assessors should be professionally trained and assessed (42). Key time points for evaluation of cancer patients are Initial diagnosis, initiation of treatment, periodic intervals during treatment, end of treatment, after treatment or transition to survival, relapse or progression, advanced disease, death, and during individual transition or reassessment (43). At present, there are many kinds of meaning in life scales used at home and abroad and they have not been unified. The main meaning life scales used abroad includes the Meaning sub-scale, the Quality of Life at the End of Life Scale, the Purpose of Life Scale, the Life Orientation Test using the LOT Scale, the Crumbaugh Scale, the sub-scale of the MC Gill Quality of Life Questionnaire, and the Attitude Toward Life Scale (23, 31). The life meaning scale used in China mainly includes the Life attitude Profile scale adapted by Taiwan scholar He Yingqi (37), meaning in Life scale for Advanced Cancer Patients developed by Wu Yongsheng (27, 29). and Chinese Version of meaning in Life Scale for Cancer Patients developed by XIA (47). According to literature review and group discussion, foreign scales may not be suitable for clinical Settings in China, so they are not recommended to be used directly. In China, the life attitude profile scale is mainly used in the study of the meaning of life of students, especially college students. meaning in Life scale for Advanced Cancer Patients is the only scale measuring meaning in life of patients with advanced cancer in China at present, which is widely used in the status quo of patients with advanced cancer and the evaluation of the effect of intervention research. Chinese Version of Meaning in Life Scale is mainly used to explore the level of meaning in life and related influencing factors of different types of cancer patients. Hamilton Anxiety Scale was used for anxiety. Hamilton Depression Scale was used for depression status. The quality of life was measured by the domestic quality of life scale for cancer patients developed by Wan Chonghua et al. Each scale has different characteristics, and researchers can evaluate the choice of scale according to the purpose of the study and the actual situation of the study subjects.

### 4.5 Intervention tips and cautions

Research has shown the following considerations and tips when implementing interventions with cancer patients. The first step should be to establish a trusting relationship with the patient early in the disease and to understand the patient’s needs. Using empathetic listening skills to understand the patient’s pain, family relationships and functioning, and showing respect from the team so that the patient can open up and share their feelings (44, 48). Some studies have suggested that clinical interventions should be individualized, others have advocated group interventions for cancer populations with lower symptom burdens (26), and others have suggested that psycho-social interventions for patients and their families are more effective than individual interventions (42). Secondly the search for the meaning of life can be better facilitated by combining multiple modalities in a rational way. Teskereci et al. suggest that combining humanism and existentialism in the care of oncology patients may increase hope and meaning in the lives of gynecologic cancer patients (31). Gomez-Batiste et al. noted that integrating psycho-social support with existing outpatient palliative care services is also a proven model (41). Research suggests that the number of interventions is also critical, with a minimum of three interventions required for maximum benefit (26, 41). Also the long term impact of the intervention on the patient needs to be considered, especially for those who are long term survivors of cancer (26). Therefore in clinical work, medical staff should develop specific intervention models based on the combination of assessment results and the actual situation. This study has also some limitations. First, most of the included literature came from abroad, which may be different from the domestic in terms of cultural background and healthcare system, and the clinical application needs to consider the adaptability problem carefully. Second, due to limitations in the scope of the study, only the evidence was summarized and the effect sizes of the interventions were not analyzed, suggesting that future studies be conducted. In this study, an evidence-based evidence on meaning-of-life interventions for cancer patients was provided through a rigorous and scientific process of literature search and screening, literature quality evaluation and evidence aggregation. However, there are no clear findings on the effect size of meaning in life after intervention. In the future, more in-depth research is necessary to enrich the content of evidence and provide high-quality guidance for clinical practice. Therefore, it is recommended for nurses to use the best evidence combined with their own clinical experience, patient needs, and actual clinical scenarios, to develop individualized care and management plan and ultimately realize the transformation of the best evidence.

## 5 Conclusion

This study summarizes the evidence of meaning in life interventions for cancer patients, and provides a reference for improving the mental and psychological health of cancer patients. When applying the evidence, healthcare staff should provide personalized measures according to the symptoms and needs of patients.
